# News and Events of the Week

**Published:** 1909-10-23

**Authors:** 


### News and Events of the Week.

The autumn meetings of the Women's Total Abstinence Union were held in Huddersfield from October 9 to 13, and were most successful.

At a recent meeting of the Ardrossan School Board it was reported that Dr. C. R. M'Donald, medical officer for Saltcoats, had appeared at the school for the purpose of inspecting it, but that the headmaster had declined to admit him, having received no instructions from the School Board. The meeting approved of the action of the headmaster. The medical officer visited the school at the request of the Town Council.

Dr. Charles Burland, the new senior medical inspector of the Board of Trade, is an old student of Guy's Hospital, and graduated at the University of London with first-class honours, and a scholarship in forensic medicine. After holding junior staff appointments at St. Mary's and the Children's Hospital in Great Ormonde Street, he joined the Cunard Line, and in 1882 was appointed medical officer by the Board of Trade at Queenstown. He has served successively in that capacity in the ports of Liverpool and Glasgow. He has contributed articles upon "Hygiene and first-aid in the merchant service" and upon "Nautical hygiene, with special reference to cholera."

In the course of an address on the Poor Law Problem last week, Dr. T. J. Macnamara, M.P., said that in England and Wales alone there were 900,000 persons whose condition called for public assistance. One in eight was insane, and many were physically afflicted. Roughly, two out of every three were given outdoor relief, and the third was maintained in some form of institution. The total annual expenditure of public money on Poor Law relief was, as near as possible, £14,000,000, of which £3,770,000 was spent in London. Indoor relief cost London £2,500,000, and outdoor relief about £400,000. In 1850, in England and Wales, the number of persons in receipt of Poor Law relief, exclusive of insane and casuals, was 56.5 per 1,000 of the population; and in 1908, 22.1. The figures for London were: 1850, 45.9; and in 1908, 24.4. In 1890 it had fallen to 21.8. Every class of pauper had decreased, with the exception of the insane poor and the vagrant or casual poor.

Professor Osler formally opened three new laboratories for physiology, chemistry, and physics at the London Hospital Medical College on Friday, the 15th inst. Mr. Sydney Holland, the chairman of the hospital, the Vice-Chancellor, and Professor H. A. Miers, with the staff and lecturers of the hospital, received Professor Osler in the warden's room, and before the ceremony conducted him through the new laboratories. These have been constructed and equipped at a cost of about £8,000, which has been borrowed at 4½ per cent. interest. The new laboratories afford accommodation for about 120 students. Professor Osler, in declaring the laboratories open, referred to the question of a degree for London students, and said that it was a scandalous injustice that the London student could not, by a good ordinary examination, get a good ordinary, honest M.D. degree. The University of London had failed, the hospitals had failed, and the conjoint board had failed. They must, therefore, help themselves and organise. If London medical students were to combine they could get it in a year or two. The body of medical students in London was large and powerful enough to organise and agitate and to get what they wanted.

The Kingston Town Council, at a recent meeting held *in camera*, decided to reduce the salary of their medical officer of health (Dr. H. Beale Collins) by the sum of £55 a year. No public explanation has been offered as to this unusual procedure, which has evoked very strong comment in the town, and a proposal has been started to promote a public testimonial to Dr. Collins in recognition of his 16 years' service.

The public introductory address of the Honyman Gillespie Post-graduate Lecture Courses on Homœopathy will be delivered by Dr. George Burford on "The Medicine of the Future: Coming Events that cast their Shadows before," at the London Homœopathic Hospital, Great Ormond Street, W.C., at 5 p.m., on Thursday, October 28, 1909. The chair will be taken by the Lady Mayoress, who will be supported by the Lord Mayor, Sir George Wyatt Truscott, Bart.

At a recent meeting of the Cardiff School Management Committee the medical officer submitted a report as to the results of the medical inspection. Altogether 4,172 children were examined, 4,031 at the school and 141 at the City Hall. Of the 4,031, 811, or 20 per cent., were found to require medical treatment or supervision, and parents were notified. The principal defects found among the girls were as follow: 6.2 per cent. suffered from defective vision, 0.9 per cent. from diseases of the ear, 0.7 infectious skin diseases, 0.9 tuberculosis, and 14.8 per cent. enlarged tonsils and adenoids. Among the boys examined 3.5 suffered from defective vision, 1.8 diseases of the ear, 3.1 infectious skin diseases, 6.6 tuberculosis, and 16.1 enlarged tonsils and adenoids. Amongst the infants 1.2 suffered from diseases of the ear, 2.6 infectious skin diseases, 6.6 tuberculosis, and 9.4 enlarged tonsils and adenoids. These figures do not include 88.9 per cent. of the children of both sexes found with defective teeth.

The annual meeting of the Society of Medical Officers of Health was held a fortnight ago, at the offices, 1 Upper Montague Street, W.C. The newly elected President, Dr. H. Cooper Pattin (Norwich), was inducted to the chair, and there was a large attendance. On behalf of the Scottish Branch, Dr. Armstrong presented the Society with a portrait of Dr. J. McVail. The annual report stated that the membership now numbers 976, as against 387 in 1891. Dr. R. Dudfield had been appointed a representative of the Society on the Sanitary Inspectors Examination Board. The Council had made representation to the Local Government Board in favour of a quinquennial census and also a uniform system of keeping the accounts of isolation hospitals by sanitary authorities. The Council also stated that they had decided not to take any official part in the work of the Society for Breaking up the Poor Law. The report was adopted and all the officers were re-elected. The President delivered his inaugural address. In the evening the annual dinner of the Society was held at the Criterion Restaurant. The Dean of Norwich proposed the toast of "Health and Education Authorities," for whom the Deputy Mayor of Norwich and Mr. Sidney Webb replied. Sir Robert Morant (Secretary of the Board of Education) proposed the toast of the evening, and Dr. Sergeant gave "Kindred Societies," for whom Sir James Crichton-Browne, Surgeon-General Evatt, C.B., and Dr. A. Newsholme replied.

A vacancy in the office of surgeon at St. Bartholomew's Hospital has been created by the resignation of Mr. W. Harrison Cripps, F.R.C.S. Mr. Harrison Cripps has been appointed a governor of the hospital on his resignation.

After the opening of the new laboratories at the London Hospital, Professor Osler delivered the first "Schorstein" lecture in the library. The friends of the late Dr. Schorstein have raised a fund, which amounts to £1,010, to endow a lectureship in clinical medicine in his memory.

It has been determined that at the expiration of five years' service the assistant physicians and assistant surgeons at St. Bartholomew's Hospital, including those attached to special departments, will be given the title of physician or surgeon with charge of out-patients.

The following honorary appointments have been made at the Royal Eye Hospital, Southwark, S.E.: Mr. James Collier, F.R.C.P., honorary physician; Mr. W. M. Bergin, F.R.C.S., an honorary assistant surgeon; Sir W. J. Collins, M.D., M.P., senior honorary surgeon, vice Professor M. McHardy, resigned. Mr. Malcolm McHardy, F.R.C.S., senior ophthalmic surgeon, King's College Hospital, and Professor of Ophthalmology, King's College, London, has been appointed honorary consulting surgeon to the hospital.

The American Ambassador will preside at the opening meeting of the winter session of the London School of Tropical Medicine, Albert Docks, which is to be inaugurated on the 26th inst. by an address by Professor W. Osler, M.D., F.R.S., Regius Professor of Medicine at Oxford University, on "The Nation and the Tropics." In the evening of the same day a dinner of the staff and students of the school will be held at the Savoy Hotel, under the presidency of Dr. Bagshawe, the Director of the Sleeping Sickness Commission, an old student of the school.

Professor Theodore Schott, of Nauheim, opened the session of the Postgraduate College, West London Hospital, on Monday, October 11, with an address entitled "A Renewed Research on the Subject of Acute Overstrain of the Heart." In the course of it he pointed out the increased attention given during the last fifty years to functional as opposed to valvular derangements, and said that it has been physiologically established that the simple performance of labour can lead to dilatation, and that there is such a thing as pericardiac hypertrophy. He also referred to the exaggerated pursuit of athletics, with the number of heart cases occurring in young men who were otherwise free from disease, and unweakened by immoderate drinking and smoking.

At the quarterly meeting of the Council of the Royal College of Surgeons last week a resolution was passed recording the Council's deep regret at the death of Sir Thomas Smith, and expressing sympathy with his family. A report was received from the president and vice-presidents of the College Dental Charter on the proposed celebration of the jubilee of the grant of the charter enabling the college to confer diplomas in dental surgery, and it was decided that the celebration should take the form of a dinner given by the college to the leading members of the dental profession at the college on December 2. A letter was read from the joint secretaries to the Royal Commission on University Education in London, stating that the Commission will be prepared to receive information upon the views of the Royal College of Surgeons on questions connected with medical education in London.

The honorary secretaries of King Edward's Hospital Fund for London have received a cheque for £4,775 from Sir Charles Hardinge, being part of the surplus remaining after the liquidation of the Franco-British Exhibition. A cheque for £4,775 has also been forwarded to the French Ambassador for distribution to charitable French institutions.

